# Salt glands of recretohalophyte *Tamarix* under salinity: Their evolution and adaptation

**DOI:** 10.1002/ece3.6625

**Published:** 2020-08-11

**Authors:** Xiaocen Wei, Xin Yan, Zhen Yang, Guoliang Han, Lei Wang, Fang Yuan, Baoshan Wang

**Affiliations:** ^1^ Shandong Provincial Key Laboratory of Plant Stress College of Life Sciences Shandong Normal University Ji'nan China; ^2^ Shandong Provincial Key Laboratory of Microbial Engineering School of Biologic Engineering Qilu University of Technology (Shandong Academy of Sciences) Ji'nan China

**Keywords:** evolution, phylogenetic analysis, salt glands, salt tolerance, *Tamarix*

## Abstract

Here, we studied the evolution of salt glands in 11 species of *Tamarix* and determined their role in adaptation to saline environments by measuring the effect of NaCl on plant growth and salt gland characteristics. Cluster analysis divided *Tamarix* species into three types (types I–III) according to salt‐gland characteristics. A phylogenetic tree based on ITS sequences indicated an evolutionary relationship consistent with the geographical distribution of *Tamarix*. We measured growth under different NaCl conditions (0, 100, 200, and 300 mM) for 40 days in three species (*T*. *gallica*, *T*. *ramosissima,* and *T*. *laxa*) representing the three *Tamarix* types. With increasing NaCl concentration, the biomass of all species was significantly reduced, especially that of *T*. *gallica*. Salt secretion ability and salt‐gland density showed similar trends in three types. The order of salt tolerance was type I > type II > type III. We conclude that during *Tamarix* adaptation to salinity, salt‐gland evolution followed two directions: one increasing salt‐gland density, and the other increasing salt secretion rate per salt‐gland. This study provides a basis for potential mechanisms of recretohalophyte adaptation to salinity.

## Graphical abstract

In conclusion, *Tamarix* species can be divided into three types demonstrating salt tolerance in the order type I > type II > type III; this was confirmed by phylogenetic relationships based on ITS sequences. Salt‐gland evolution has occurred in two directions: one to increase the density of salt glands under low salinity environments, and the other to increase salt secretion rate per salt gland under high salinity environments. However, more research must be conducted using specific molecular markers for salt glands to understand the most reasonable evolution of salt glands.

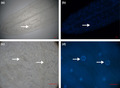

## 1 INTRODUCTION

More than 800 million hectares of land worldwide is salt‐affected, and although high levels of salt generally reduce plant growth, tolerance to soil salinity differs greatly among plant species (Munns & Tester, 2008; Takemura et al., 2000; Tan, Lin, Lim, Kumar, & Loh, 2013). With increasing demand for agricultural products and continued spread of salt‐affected soils, understanding plant evolution and adaptation to salinity and how to develop crops suitable for saline environments is increasingly important (Rozema & Flowers, 2008; Zhu, 2002, 2016).

Halophytes can survive and reproduce in environments with 200 mM NaCl or more (Flowers & Colmer, 2008). Most species of *Tamarix* are typical exo‐recretohalophytes belonging to the family Tamaricaceae and possess typical salt excretory structures called salt glands distributed throughout the epidermis of leaves and branches. The salt gland of *Tamarix* is a complex of eight cells: two inner collecting cells and six outer secretory cells (Bosabalidis, 2010, 2012; Campbell & Strong, 1964). Salt glands actively secrete excessive ions out of plant cells to avoid toxicity (Yuan, Leng, & Wang, 2016); however, there are major differences in both the density and secretion rate of salt glands among species. For instance, after increasing soil salinity, secretory activity of salt glands of *Tamarix ramosissima* and *Tamarix laxa* significantly increased, and the secretion of Na^+^ far exceeded the secretion of K^+^ (Ma, Tian, Feng, & Yuan, 2011).

Evolutionary studies of *Tamarix* beginning in the 1960s have mainly focused on pollen, stamens, and seed. Through observation of stamens and flower discs, Zohary and Baum (1965) deduced that the stamen was polyandrous and that two whorls in the most primitive species of *Tamarix* eventually evolved into a single ring through filament fusion and degradation. Baum, Bassett, and Crompton (1970); Baum, Bassett, and Crompton (1971) classified *Tamarix* pollen into three types based on reticulate ornamentation of the extine—crude reticulate, medium reticulate, and fine reticulate—suggesting that pollen evolved from crude reticulate to fine reticulate. Light and scanning electron microscopy of *Tamarix* seeds revealed that the whole surface of primitive seeds is covered with hair, while other seeds only have hair distributed on the top as an adaptation to the changing moisture in the living environment (Zhang, Pan, & Yin, 1998). Primitive species lack papillae or epidermal hairs compared with evolved species (Zhang et al., 2018). However, little is known about the evolution of salt glands and their role in salinity adaptation of different species of *Tamarix*.

Species of *Tamarix* are distributed widely, from coastal to inland areas, and have adapted to changed environments. As the most sensitive organs to environmental change, leaves contain abundant evolutionary information, especially in the form of specialized structures such as salt glands (Conover, 1991; Jones, 1986). The mechanism of salt tolerance is a topic worth studying (Flowers, Galal, & Bromham, 2010). Early research on the salt glands of *Tamarix* mainly focused on their structure and salt secretion physiology, and rarely on their evolution. In the present study, we aimed to understand the evolution of *Tamarix* salt glands through scientific classification and comparison of salt secretion responses to different NaCl concentrations in different species. Our results improve our understanding of the evolution of *Tamarix* salt glands for adaptation to saline environments.

## 2 MATERIALS AND METHODS

### 2.1 Plant material and culture conditions

Branches from plants of 11 species of *Tamarix* showing similar growth were obtained from saline land of the Yellow River Delta, China (N37°25′; E118°58′) in 2018: *Tamarix laxa* Willd, *Tamarix elongata* Ldb., *Tamarix ramosissima* Ldb., *Tamarix gansuensis* H. Z. Zhang, *Tamarix hispida* Willd, *Tamarix chinensis* Lour., *Tamarix hohenackeri* Bge., *Tamarix gallica*, *Tamarix leptostachys* Bge., *Tamarix arceuthoides* Bge., and *Tamarix austromongolica* Nakai.

For classification, branches were cut into 10‐cm pieces, soaked in water for 24 hr and then disinfected in 0.5% potassium permanganate solution for 30 min followed by washing with tap water. The cuttings were planted in a mixture of sand and soil (1:4, v/v) for rooting and sprouting. After sprouting, the cuttings were sprayed with water every 3 days. Light intensity is about 600 µmol m^−2^ s^−1^.The length of day and night is about 14 hr/10 hr. Temperature in the greenhouse was 30°C/20°C (day/night), and relative humidity was maintained at about 75%. Cuttings were cultured for 90 days.

In the following section, the leaves of different *Tamarix* were drawn to investigate the salt gland density and salt secretion rate. And based on the two data, three types were classified according to the cluster and a phylogenetic tree, and then physiological indicators were measured in the three types.

### 2.2 Measurement of density and diameter of salt glands

Assimilation branches were recognized as that the leaves were covered with thick cuticles and highly developed chloroplast existed, common in some drought tolerance species such as *Elaeagnus angustifolia* and *Tamarix* (Cheng‐lee & Rong‐ao, 1981). Mature leaves in the middle third of assimilation branches of different species were taken and were immersed in a solution containing ethanol and acetic acid (3:1, v/v) under vacuum for 1 hr (Kuwabara & Nagata, 2006). Samples of shoots were taken in the same year. The leaves were taken out and placed on a glass slide for 2 hr being treated with the lactic acid saturated with chloral hydrate described by Lux, Morita, Abe, and Ito (2005), then covered with a cover glass for observation.

A differential interference contrast (DIC) microscope (ECLIPSE 80i, Nikon, Japan) was used to determine density and diameter of salt glands under 330–380 nm UV excitation (Yuan, Chen, Leng, & Wang, 2013). These were counted at ×400 magnification in 30 fields selected randomly according to the method of Liu and Meinke (1998). Density of the salt glands is obtained by taking the average of 10 fields of view in 5 replicates according to the method of Ding, Chen, Sui, and Wang (2010) and Leng, Yuan, Dong, Wang, and Wang (2018).

### 2.3 Determination of salt secretion rate

The cut ends of branches were immersed in deionized water or 100 mM NaCl for 24 hr (Semenova, Fomina, & Biel, 2010). Before treatment, the branches were washed with water to rinse off the salt that has been secreted on the surface. The bottom of each branch was removed, and the remainder (leaves) was immediately placed in a test tube with 10 ml of deionized water and shaken for 20 s to wash the salt from the leaves for measuring ion content (Drennan & Pammenter, 1982). Na^+^ concentration was measured using an ion chromatograph (DIONEX ICS‐1100, Thermo, USA). Each excised branch was then washed with deionized water, quickly dried with absorbent paper and weighed. Salt secretion rate of total salt glands was calculated using the following formula.

$$\text{Secretion}\;\text{rate}\;\left(\times 10^{‐3}\;\text{mM}\;{\text{Na}}^{+}{\left[g\;\;\text{fresh}\;\text{weight}\right]}^{‐1}\;{\text{hr}}^{‐1}\right)={\text{Na}}^{+}\;\text{secretion}\;\text{quantity}/\left(\text{time}\times \text{fresh}\;\text{weight}\times 23\right)$$

One leaf was removed, and the concentration of Na^+^/K^+^/Mg^2^^+^/Ca^2+^ was measured as above. The leaf was made into a temporary film according to the above method, and the number of all salt glands on the leaf was counted. Salt secretion rate per salt gland was calculated using the following formula.

$$\begin{array}{c} \text{Secretion}\;\text{rate}\;\text{per}\;\text{salt}\;\text{gland}\;(\times 10^{‐6}\;\text{mM}/\text{day})\\ =\text{ion}\;\text{secretion}\;\text{quantity}/(\text{time}\times \text{total}\;\text{number}\;\text{of}\;\text{salt}\;\text{glands}\times \text{the}\;\text{relative}\;\text{molecular}\;\text{mass}\;\text{of}\;\text{the}\;\text{corresponding}\;\text{ion}) \end{array}$$

### 2.4 Clustering based on salt‐gland characteristics

Cluster analysis according to salt‐gland characteristics was performed using SPSS 22 software (SPSS Software Inc.) based on Euclidean distance using the most long‐distance method (Wu et al., 2019).

### 2.5 Reconstruction of a phylogenetic tree

Internal transcribed spacer (ITS) sequences of the 18S–5.8S–28S nuclear ribosomal cistrons of 31 *Tamarix* species were obtained from NCBI GenBank. A phylogenetic tree was reconstructed using the maximum‐likelihood (ML) method by MEGA 5.0 with *Reaumuria soongarica* as an outgroup (Alvarez & Wendel, 2003; Bailey, Carr, Harris, Hughes, & Evolution, 2003; Erdogan & Mehlenbacher, 2000; Gregory, 2008).

### 2.6 Determination of physiological parameters

Three species (*T*. *gallica*, *T*. *ramosissima*, and *T*. *laxa*) representing different clusters in the phylogenetic analysis were used for salt stress experiments. Branches were cut into 8‐cm‐long pieces and planted in sand. After sprouting, the plants were watered with Hoagland's solution every 3 days. After 40 days, plants were watered with Hoagland's solution containing different concentrations of NaCl (0, 100, 200, and 300 mM) for a further 40 days. To avoid salt shock, the salt concentration was increased daily in increments of 100 mM NaCl to the desired level. Plants were grown in a greenhouse as described above.

Plants were separated into roots and shoots. The length of the main stem was measured with a ruler to determine plant height. Stems were then washed three times with ultrapure water, quickly dried with blotting paper, and weighed to determine fresh weight (FW). Samples were dried at 105°C for 10 min and then maintained at 70°C until reaching a constant weight before weighing to determine dry weight (DW). Area of leaves from the same leaf position was determined under a light microscope.

Salt‐gland density and diameter, salt secretion rate and Na^+^ concentrations of the three species were determined as described above.

### 2.7 Determination of lipid peroxidation level

Malondialdehyde (MDA) content was determined using the 2‐thiobarbituric acid (TBA) assay described by Draper and Hadley (1990) with some modifications. In brief, leaves (0.4 g FW) were extracted in 5 ml of solution containing 0.1% trichloroacetic acid (TCA) and 5 ml of 0.5% TBA. Extracts were boiled for 10 min and cooled in water, then centrifuged at 1,400 *g* (Eppendorf Centrifuge 5417R) for 15 min. MDA contents were calculated as μmol (g FW)^−1^ from *A*_532_ and *A*_600_ values using a molar absorption coefficient of 1.56 × 10^5^ (Yuan, Leng, et al., 2019).

### 2.8 Statistical analysis

Statistical analysis was performed with SPSS 22 software. Results were subjected to a one‐way analysis of variance (ANOVA), and Dunnett's test was used to determine significant differences between means (*p* < .05). In the figures, error bars represent means ± standard deviations and different letters indicate significant differences at *p* = .05. Figures were drawn using SigmaPlot 12.50 (Systat software; Leng et al., 2019).

## 3 RESULTS

### 3.1 Salt‐gland morphology in *Tamarix* leaves

**FIGURE 1:**
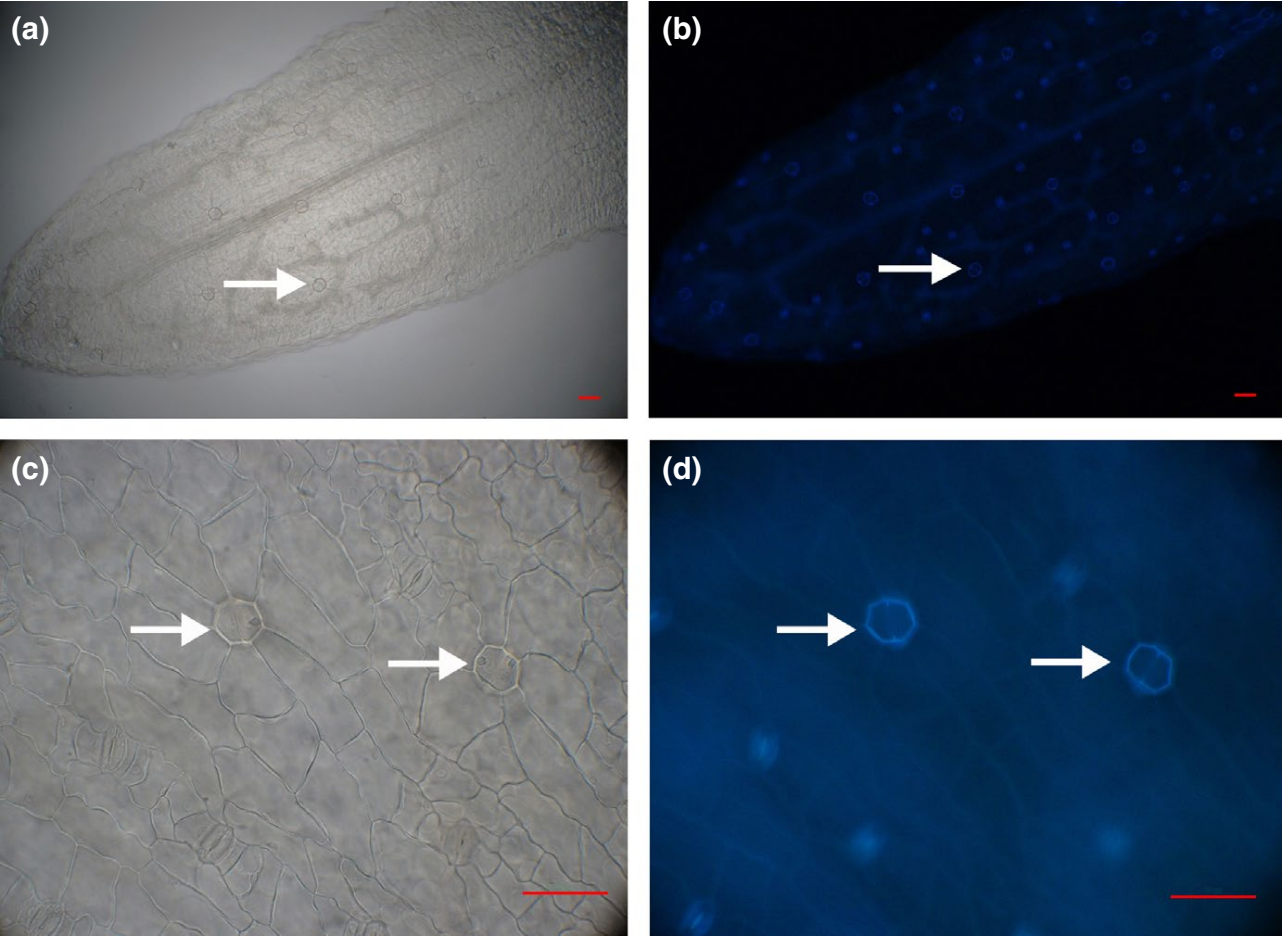
Salt glands of *T. elongata* leaf lower epidermis. DIC and fluorescence images of salt glands of a *T. elongata* leaf lower epidermis under × 100 magnification (a, b) and ×400 magnification (c, d). Bars = 100 μm (a, b); 50 μm (c, d). Arrows indicate salt glands

We observed salt glands in the epidermis of leaves and assimilating branches of all 11 species of *Tamarix* using a DIC microscope; only the top two secretory cells could be seen. Salt glands of *T*. *elongata* are shown in Figure 1 as a representative. We observed autofluorescence of cell walls in the salt‐gland cell fusion area under ultraviolet excitation at 330–380 nm (Figure 1b,d). This unique autofluorescence of the salt gland can be used as a simple and reliable tool to observe salt‐gland distribution and morphology.

### 3.2 Cluster analysis based on salt secretion capacity

**FIGURE 2:**
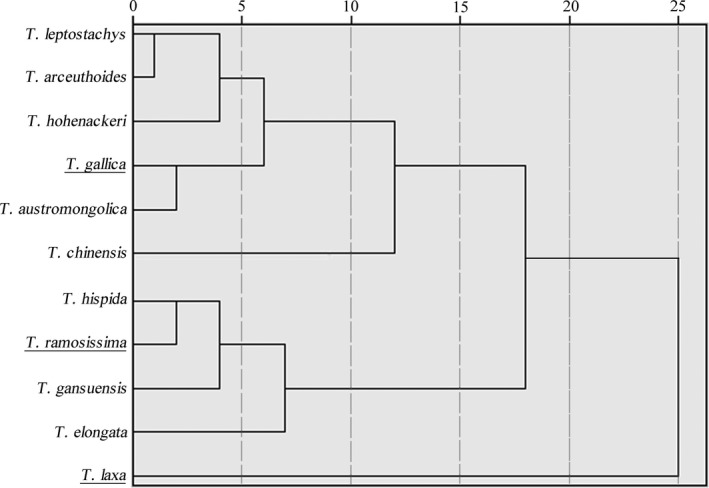
Cluster analysis of 11 species of *Tamarix* based on salt‐gland characteristics

Although salt glands of different *Tamarix* species have similar morphology and autofluorescence, salt‐gland diameter and density, total salt secretion rate and ion components of the secretion are dramatically different. We determined salt‐gland density and diameter in the upper and lower epidermis of leaves from 11 *Tamarix* species and the salt secretion rate with 0 and 100 mM NaCl (Table 1). The secretion rate for single salt gland of the 11 species under 0 and 100 mM NaCl is shown in Table 2. Cluster analysis with a Euclidean distance of 15 divided the 11 species of *Tamarix* into three types (Figure 2), and the average of each parameter about salt glands was calculated (Figure S1). We can summarize the characteristics of the three types of *Tamarix* as follows.

**TABLE 1:** Density, diameter, and secretion rate of salt glands of 11 species of *Tamarix*

*Tamarix s*pecies	Salt gland density (number mm^−2^)		Salt gland diameter (μm)		Secretion rate (×10^–3^ mM/g FW hr^−1^)	
	Upper epidermis	Lower epidermis	Upper epidermis	Lower epidermis	0	100 mmol/L NaCl
*T. chinensis*	50.96 ± 10.30b	44.59 ± 11.24cd	20.84 ± 2.99f	28.42 ± 1.93abc	0.69 ± 0.10d	7.02 ± 0.68c
*T. elongata*	37.06 ± 10.01cd	42.91 ± 12.35cd	28.60 ± 2.22abc	27.38 ± 2.23abc	0.19 ± 0.09gh	8.72 ± 0.38b
*T. leptostachys*	67.51 ± 18.73a	42.29 ± 11.19cd	23.86 ± 2.16de	29.09 ± 1.52ab	0.11 ± 0.02h	8.96 ± 1.23b
*T. arceuthoides*	43.68 ± 9.71bc	38.13 ± 7.25d	22.33 ± 2.93ef	29.05 ± 2.09ab	0.31 ± 0.06efg	5.38 ± 0.51d
*T. laxa*	51.62 ± 17.67b	73.61 ± 18.99a	23.07 ± 3.21def	27.18 ± 1.62d	3.34 ± 0.16a	21.87 ± 0.88a
*T. gallica*	51.62 ± 18.83b	39.31 ± 10.67d	27.69 ± 1.48ab	27.98 ± 1.39bc	0.44 ± 0.09e	1.01 ± 0.11h
*T. hispida*	29.12 ± 11.28de	51.31 ± 15.00c	29.61 ± 3.47a	29.57 ± 1.92a	0.84 ± 0.05c	8.99 ± 0.97b
*T. gansuensis*	21.18 ± 7.60e	28.43 ± 10.77e	25.21 ± 1.63bcd	28.85 ± 1.69abc	0.44 ± 0.11e	6.04 ± 0.75cd
*T. austromongolica*	48.98 ± 14.95bc	38.13 ± 7.25d	21.04 ± 1.07f	27.40 ± 1.83cd	0.24 ± 0.02fgh	3.05 ± 0.44ef
*T. ramosissima*	21.84 ± 7.67e	29.81 ± 11.72e	27.29 ± 4.22abc	29.27 ± 1.93ab	0.37 ± 0.06ef	1.81 ± 0.25gh
*T. hohenackeri*	52.95 ± 18.73b	63.34 ± 13.59b	24.98 ± 2.55cd	27.5 0 ± 1.77cd	1.11 ± 0.12b	3.73 ± 0.10e

Note

Data are average of three replicates ± *SD*; Different letters in the same column indicate significant difference at *p* = .05.

**TABLE 2:** Secretion rate per salt gland for 11 *Tamarix* species

*Tamarix* species	Secretion rate per salt gland of leaves in cuttings immersed in deionized water (×10^–8^ mM day^−1^)				Secretion rate per salt gland of leaves in cuttings immersed in 100 mmol/L NaCl (×10^–8^ mM day^−1^)			
	Na^+^	K^+^	Mg^2+^	Ca^2+^	Na^+^	K^+^	Mg^2+^	Ca^2+^
*T. chinensis*	31.35 ± 2.39e	15.05 ± 1.41d	13.13 ± 0.42d	18.00 ± 1.30de	109.30 ± 12.83a	52.62 ± 6.49a	7.96 ± 0.50de	20.70 ± 3.35e
*T. elongata*	96.48 ± 3.39a	26.21 ± 3.36c	31.25 ± 5.42b	54.65 ± 4.58a	110.74 ± 12.04a	18.82 ± 4.49ef	37.63 ± 3.13a	62.70 ± 1.95a
*T. leptostachys*	42.96 ± 6.48d	19.33 ± 2.69cd	10.13 ± 1.00de	19.23 ± 0.45d	88.00 ± 12.48bc	22.79 ± 3.69de	9.67 ± 1.38cde	14.35 ± 2.28e
*T. arceuthoides*	47.09 ± 8.57d	15.97 ± 0.87d	11.79 ± 1.33de	28.18 ± 3.90c	70.26 ± 12.61d	17.59 ± 3.03ef	14.96 ± 2.17c	33.43 ± 4.40cd
*T. laxa*	14.61 ± 1.26f	6.10 ± 0.49e	5.38 ± 0.63f	6.78 ± 1.18f	41.35 ± 1.39e	5.41 ± 1.69h	4.75 ± 1.29e	6.23 ± 0.55f
*T. gallica*	18.61 ± 3.39f	8.79 ± 0.54e	12.00 ± 1.29de	13.65 ± 2.53e	38.09 ± 4.74e	8.77 ± 1.97gh	11.21 ± 2.83cd	9.20 ± 0.45f
*T. hispida*	65.52 ± 2.48c	30.95 ± 5.03b	29.71 ± 4.75b	43.83 ± 1.23b	109.30 ± 3.91a	21.82 ± 1.00de	30.58 ± 0.33b	33.95 ± 5.83cd
*T. gansuensis*	65.83 ± 7.87c	22.79 ± 1.82c	50.92 ± 4.88a	51.00 ± 1.18a	96.22 ± 8.04ab	26.79 ± 5.74d	32.83 ± 4.67ab	41.43 ± 5.05b
*T. austromongolica*	33.13 ± 6.43e	18.74 ± 1.87cd	7.88 ± 0.75ef	15.08 ± 1.50de	62.70 ± 7.04d	14.74 ± 3.56fg	7.21 ± 0.96de	14.58 ± 0.93e
T. *ramosissima*	66.87 ± 10.91b	31.46 ± 3.90b	19.42 ± 2.29c	41.43 ± 2.53b	108.09 ± 9.13bc	44.69 ± 3.79b	28.58 ± 4.42b	43.25 ± 3.28b
*T. hohenackeri*	46.04 ± 1.04d	21.54 ± 6.15cd	20.38 ± 1.63c	28.78 ± 6.48c	77.17 ± 6.83cd	19.82 ± 0.87ef	12.50 ± 5.13cd	30.25 ± 4.40d

Note

Data are average of 10 replicates ± *SD*; Different letters in the same column indicate significant difference at *p* = .05.

Type I: High salt gland density, low secretion rate per salt gland, high total salt secretion rate; includes *T*. *laxa*, which is naturally distributed in regions of high soil salinization and saline lakes, river terraces and desert dune edges. Type II: low salt gland density, high secretion rate per salt gland, high total salt secretion rate; includes *T*. *elongata*, *T*. *ramosissima*, *T*. *gansuensis* and *T*. *hispida*, which are naturally distributed in alluvial plains, lake edges, river terraces and wet alkali land of varying saline degree. Type III: salt‐gland density and secretion rate per salt gland between those of Type I and Type II, low total salt secretion rate; includes *T*. *chinensis*, *T*. *austromongolica*, *T*. *hohenackeri*, *T*. *gallica*, *T*. *leptostachys,* and *T*. *arceuthoides*, which are naturally distributed in mildly saline valley terraces, floodplains, rivers in mountain valleys and sandy edges of desert valleys.

### 3.3 *Tamarix* phylogenetic tree based on ITS sequences

**FIGURE 3:**
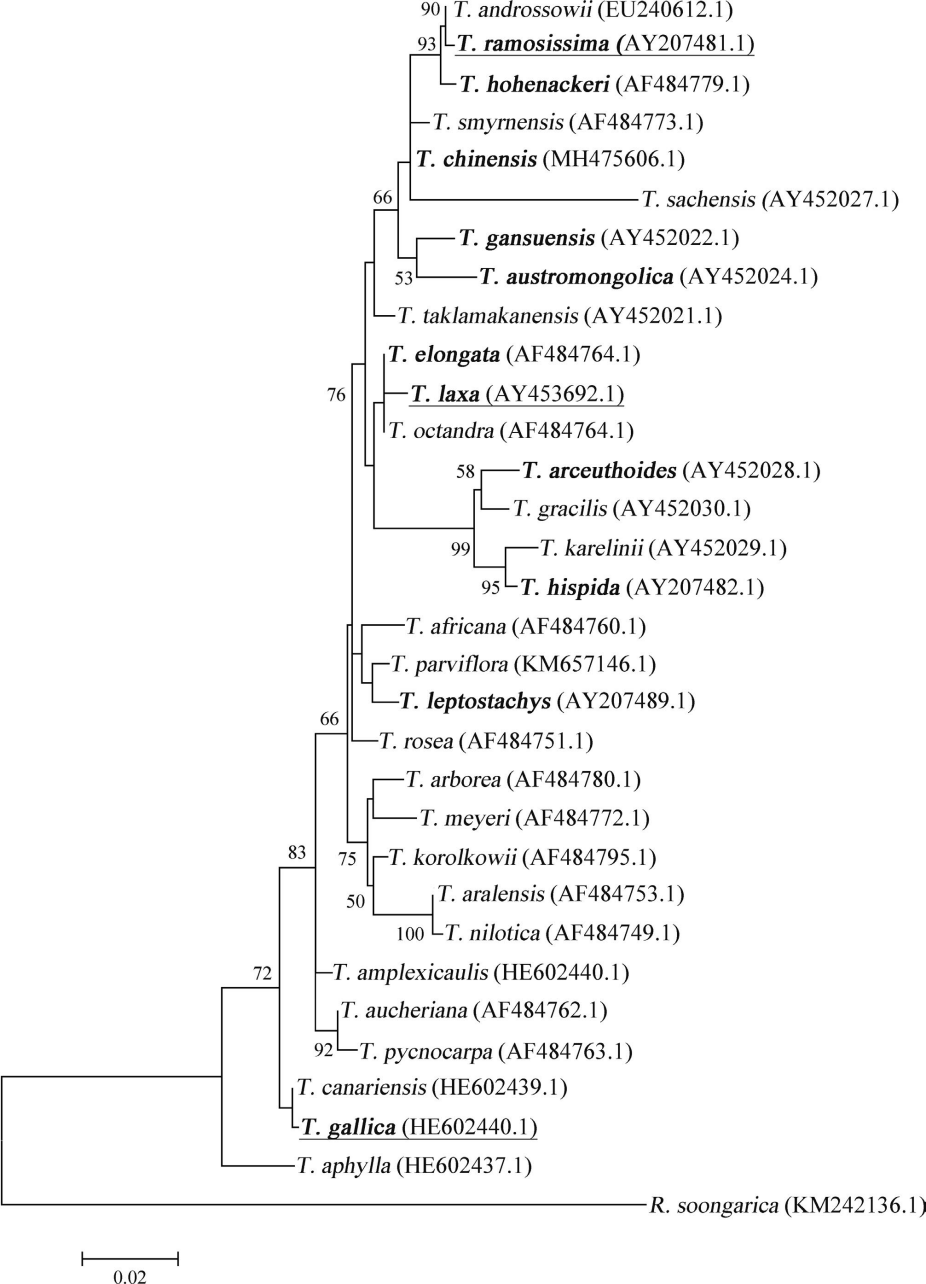
Phylogenetic tree based on ITS gene sequences of *Tamarix* species with *R. soongarica* as an outgroup

The phylogenetic tree based on ITS sequences grouped 31 *Tamarix* species according to geographical distribution (Figure 3). Most species in the upper branches were endemic to or widespread in China, indicating that the phylogenetic tree could reflect the adaptation of different species to local environments. *T*. *ramosissima*, *T*. *hohenackeri*, *T*. *chinensis*, *T*. *austromongolica,* and *T*. *gansuensis* formed a large branch containing species that grow in mildly saline soils classified as Class II. *T*. *laxa*, *T*. *elongata*, *T*. *arceuthoides*, *T*. *leptostachys,* and *T*. *hispida* were clustered into one branch, representing species that grow in dry riverbeds, wasteland, and dunes with varying degrees salinization classified as Class I. *T*. *gallica* was introduced to coastal areas of the Mediterranean, and formed its own branch in the phylogenetic tree classified as Class III. Comparison of two clustering methods in Figure S2. Although the phylogenetic tree results were not identical to those of the cluster analysis, species groupings were broadly consistent. The phylogenetic tree fully illustrated the adaptation of *Tamarix* species to their environment.

### 3.4 Determination of plant growth indicators

We chose *T*. *gallica*, *T*. *ramosissima,* and *T*. *laxa* as representatives of the three categories of *Tamarix* based on salt‐gland characteristics and treated these with different concentrations of NaCl to explore the relationship between salt tolerance and salt glands.

**FIGURE 4:**
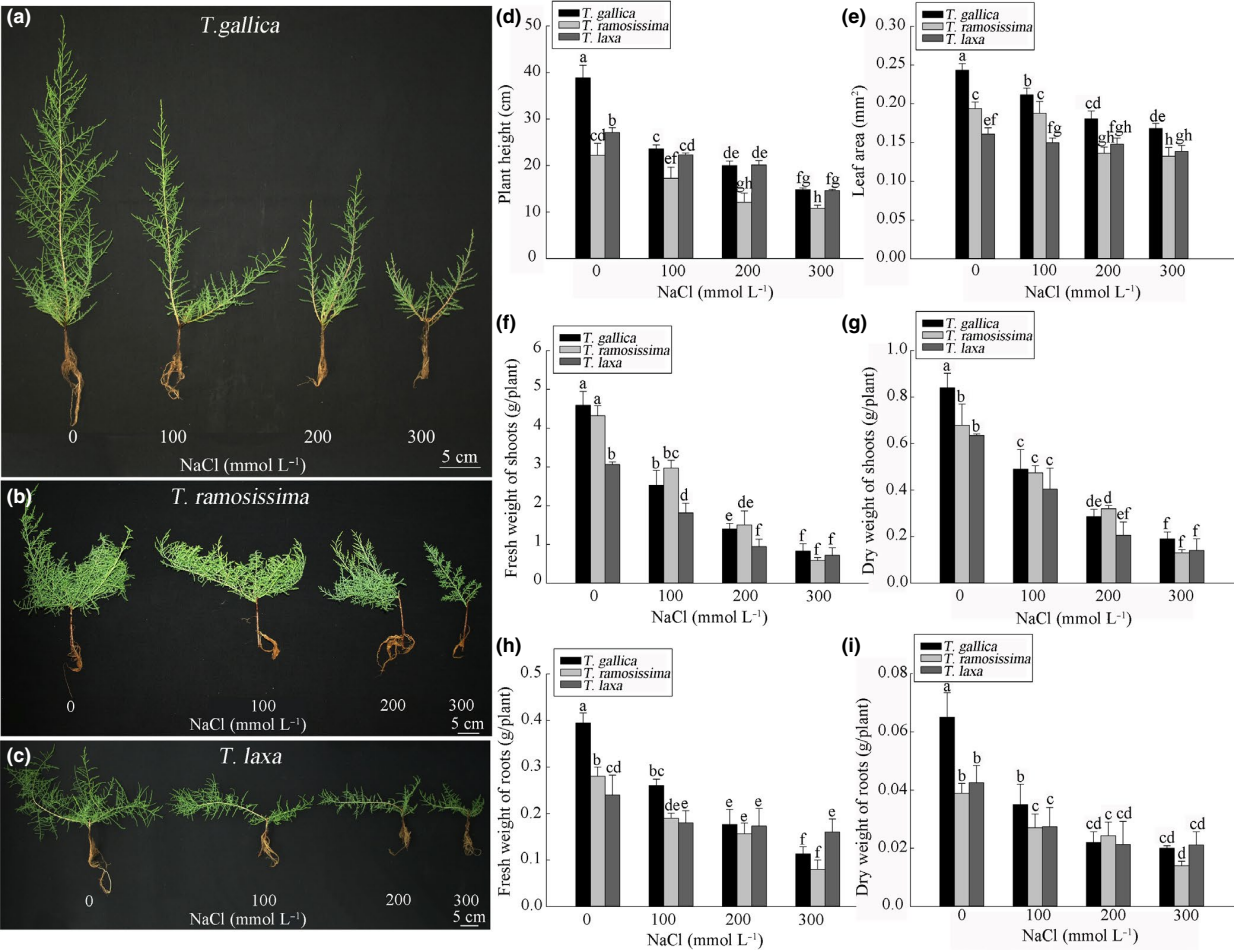
Effect of different NaCl concentrations on plant growth of *T. gallica* (a), *T. ramosissima* (b) and *T. laxa* (c) after 40 days: (d) plant height; (e) leaf area; (f) fresh weight of shoots; (g) dry weight of shoots; (h) fresh weight of roots; (i) dry weight of roots. Data are means of five replicates ±*SD*; different letters indicate significant difference at *p* = .05

Increasing NaCl concentration in the nutrient solution significantly inhibited growth of *T*. *gallica*, *T*. *ramosissima,* and *T*. *laxa* (Figure 4a–c). Moreover, old branches of *T*. *gallica* wilted, but those of *T*. *ramosissima* and *T*. *laxa* grew relatively better. As shown in Figure 4d, 100 mM NaCl significantly affected plant height of *T*. *gallica*, which was only 60% that of the control. However, after treating with 300 mM NaCl, the plant height of *T*. *laxa* dropped to 54% of the control and that of *T*. *gallica* dropped to 38% of the control.

As shown in Figure 4e, NaCl treatment had a significant effect on leaf area of *T*. *gallica*, *T*. *ramosissima,* and *T*. *laxa*. The leaf area of *T*. *gallica* was reduced significantly at 100 mM NaCl and declined to 69% of that of the control at 300 mM NaCl. There was no significant difference in leaf area between *T*. *ramosissima* treated with 100 mM NaCl and control plants, but leaf area of *T*. *ramosissima* changed sharply at 200 mM NaCl. Leaf area of *T*. *laxa* showed no significant change between NaCl concentrations, indicating that NaCl had little effect on its growth. Therefore, *T*. *laxa* was the most tolerant to NaCl, while *T*. *gallica* was the most sensitive to salt stress.

Shoot and root FW and DW were also affected by NaCl (Figure 4f–I). FW and DW of *T*. *gallica* dropped sharply compared with the control under 100 mM NaCl treatment, and those of *T*. *laxa* changed slightly. With 300 mM NaCl, shoot FW of *T*. *gallica* and *T*. *laxa* dropped to 18.0% and 23.5% of that of the control, respectively. Although 100 mM NaCl caused an expected decline in root FW and DW of *T*. *laxa* to 75% and 64% that of the control, respectively, salt concentrations greater than 100 mM NaCl led to an additional but slight decline in the root DW of *T*. *laxa*.

### 3.5 Membrane lipid peroxidation of leaves

**FIGURE 5:**
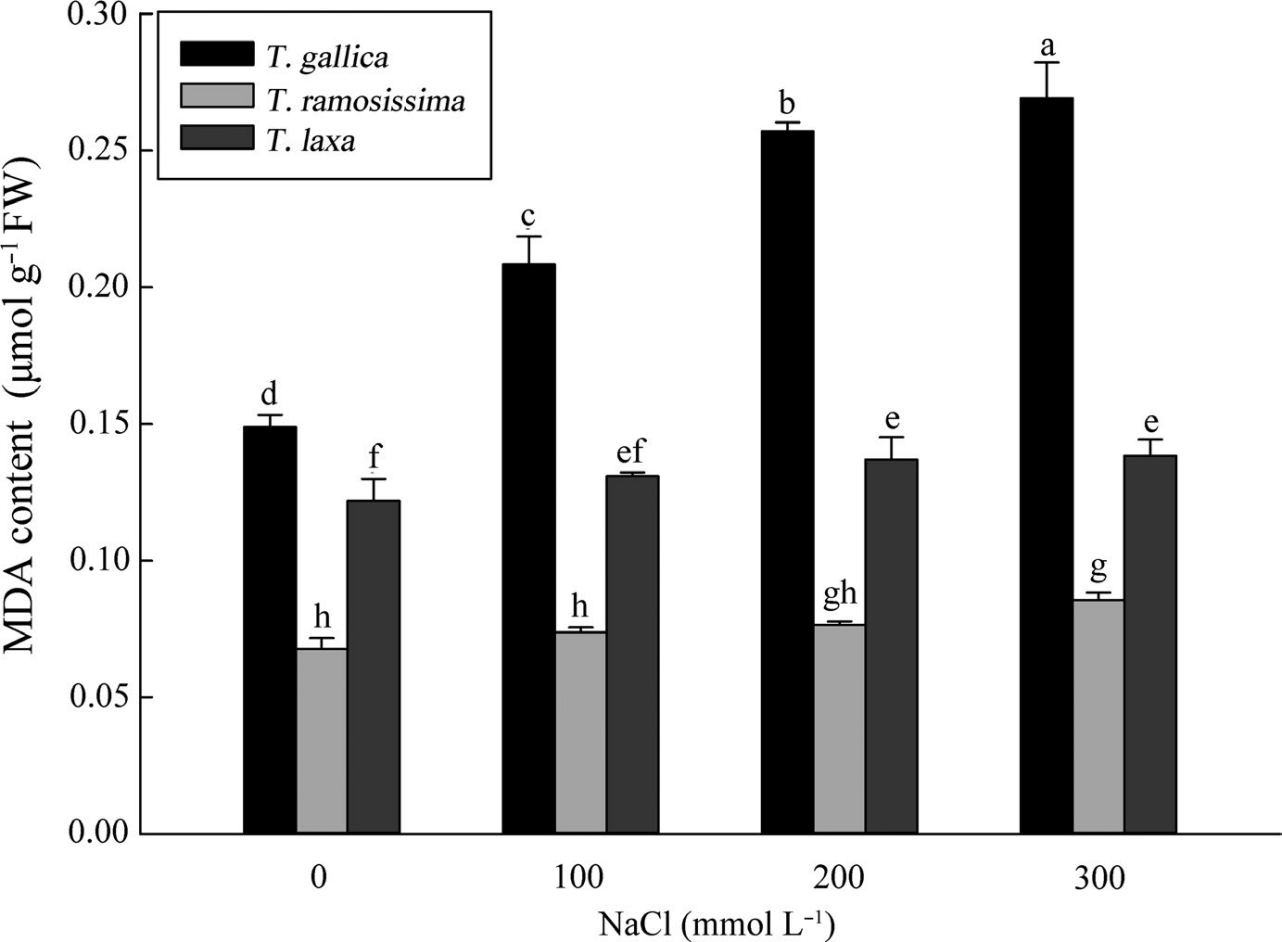
Effect of different NaCl concentrations on MDA content and plasma membrane permeability of *T. gallica*, *T. ramosissima,* and *T. laxa* after 40 days. Data are means of three replicates ±*SD*; different letters indicate significant difference at *p* = .05

We measured lipid peroxidation level in terms of MDA content (Figure 5). NaCl concentrations within the range 0–300 mM did not significantly affect MDA contents of *T*. *laxa* leaves compared with those of control plants, and the same trend was observed in *T*. *ramosissima*. Membrane permeability was enhanced with increasing NaCl concentration. The MDA content of *T*. *gallica* increased with increasing NaCl concentration. MDA content was 1.4, 1.7, and 1.8 times that of the control, respectively, with 100, 200, and 300 mM NaCl. Salt stress had adverse effects on the leaf plasma membrane, especially in *T*. *gallica*.

Plant growth parameters and lipid peroxidation results indicated that *T*. *gallica* was the most sensitive to salt stress; *T*. *laxa* was the most tolerant, and the salt tolerance of *T*. *ramosissima* was between the two. Salt tolerance of the different *Tamarix* types therefore followed the order type I > type II > type III.

### 3.6 Salt secretion by salt glands

**FIGURE 6:**
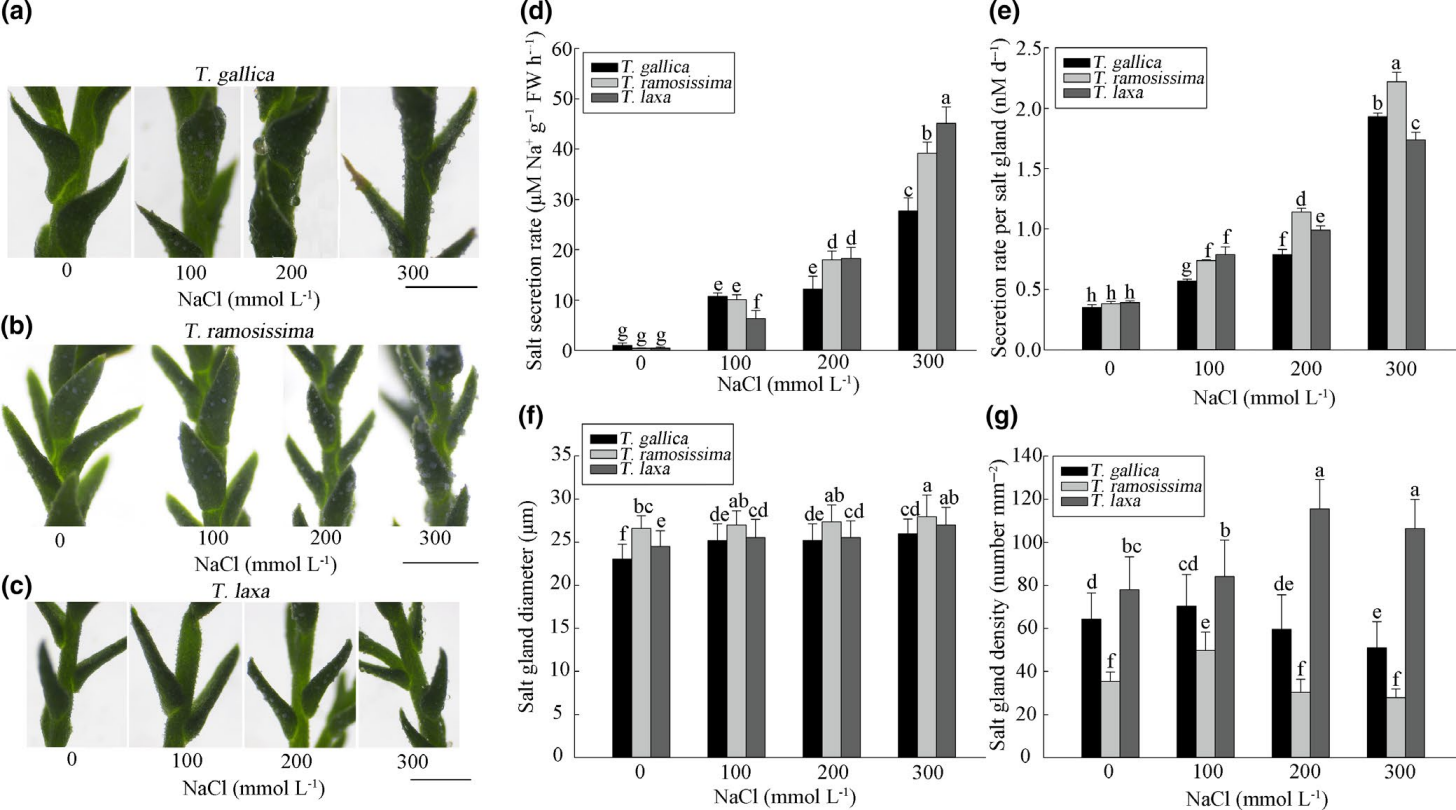
Effect of different NaCl concentrations on salt secretion by leaves and assimilating twigs of *T. gallica* (a), *T. ramosissima* (b), and *T. laxa* (c) after 40 days (×50). Effect of different NaCl concentrations on salt glands of *T. gallica*, *T. ramosissima* and *T. laxa* after 40 days: (d) salt secretion rate; (e) secretion rate per salt gland; (f) salt‐gland diameter; (g) salt‐gland density. Data are means of 5 (d), 10 (e) or 30 replicates (f, g) ±*SD*; different letters indicate significant difference at *p* = .05. Bars =  1 mm

We observed the secretion activity of salt glands of *T*. *gallica*, *T*. *ramosissima,* and *T*. *laxa* using a dissecting microscope as shown in Figure 6a–c. Salt was secreted by glands located mainly on the lower epidermis of leaves and surface of assimilating branches. Obvious salt crystallization around salt glands was apparent at 100 mM NaCl. With increasing NaCl concentration, salt crystals accumulated on the surface of *Tamarix* leaves and branches, with most seen under 300 mM NaCl treatment. Furthermore, blade tips of *T*. *gallica* were clearly yellow and wilted, indicating significant salt damage, while *T*. *ramosissima* and *T*. *laxa* grew vigorously.

Quantification of salt secretion is shown in Figure 6. The total secretion rate of *T*. *gallica*, *T*. *ramosissima,* and *T*. *laxa* salt glands increased sharply with increasing NaCl concentration, especially at 300 mM NaCl (Figure 6d), being 31, 81, and 89 times that of the control, respectively, under 300 mM NaCl treatment. The secretion rate per salt gland was also affected by NaCl (Figure 6e), with significant increases compared with the control detected at lower NaCl concentrations. Secretion rate per salt gland reached a maximum of about five times that of the control after treating with 300 mM NaCl. These results showed that *Tamarix* increased salt secretion to avoid injury in high‐salt environments, mainly through increasing the secretion rate per salt gland.

### 3.7 Salt‐gland diameter and density

Since salt glands in the lower leaf epidermis of *Tamarix* played a major role in secretion, we calculated the diameter and density of these salt glands in *T*. *gallica*, *T*. *ramosissima,* and *T*. *laxa*. The diameter of salt glands increased with increasing NaCl concentration (Figure 6f) to 1.1 times that of the control at 300 mM NaCl in all three species.

Unexpectedly, the density of salt glands in the three *Tamarix* species was higher under moderate NaCl treatment than under high NaCl treatment (Figure 6g). Salt‐gland density in *T*. *gallica* and *T*. *ramosissima* was highest under 100 mM NaCl treatment, but decreased significantly with higher concentrations of NaCl. Meanwhile, salt‐gland density of *T*. *laxa* increased gradually with NaCl treatment up to 200 mM NaCl, to a maximum of 1.5 times more than that of the control. Salt‐gland density declined slightly at 300 mM NaCl, but remained higher than that of the control.

## 4 DISCUSSION

Land plants exhibit a continuum of tolerance to Cl^‐^ and Na^+^ in their environment, from the very sensitive (e.g., *Cicer arietinum* L.; Flowers, Gaur, et al., 2010) to the tolerant (e.g., some *Tecticornia* species; English & Colmer, 2013; Yuan et al., 2016). At the upper end of tolerance for land plants is the euhalophytes, plants that can tolerate repeated exposure to seawater in the root‐zone (Breckle, 2002; Flowers & Colmer, 2008). However, halophytes represent a relatively small number of all plants, perhaps less than 1%, so tolerance to saline environments is not a fundamental trait, but one that has gradually emerged over thousands of years of evolution (Flowers & Colmer, 2015). Evolution towards more tolerance of salt stress will benefit plants.

Anatomical differences in the structure of different plants in the same environment reflect intrinsic characteristics of these species, while differences in morphology and function of the same species in different environments reflect environmental adaptability and plasticity. In the current study, we detected a significant difference in salt‐gland density, diameter, and secretion rate among 11 *Tamarix* species (Tables 1 and 2). Cluster analysis of the normalized data divided these 11 *Tamarix* species into three types with different salt secretion rates and salt‐gland density (Figure S1), which was in accordance with adaptation to their habitat. Our cluster analysis produced results similar to the classification according to characteristics of assimilating branches reported by Zhang, Tao, Zhang, and Pan (2003). The classification based on salt tolerance of domestic *Tamarix* by Zhang and Xu (1993) was also consistent with our results, with type I (*T*. *laxa*) having the highest threshold and type III being most sensitive to salt stress.

The phylogenetic tree of *Tamarix* based on ITS sequences separated the species by geographical distribution. Most species in the upper branches were endemic to or widespread in China, so the phylogenetic tree reflected the adaptation of different species to their local environment well (Figure 3). Although the phylogenetic tree results were not identical to those of cluster analysis, the species groupings were broadly consistent. Thus, the phylogenetic tree highlighted the adaptability of *Tamarix* species to saline environments and the changes in salt glands during evolution.

We further explored the relationship between salt tolerance and salt glands by treating *T*. *gallica*, *T*. *ramosissima,* and *T*. *laxa*, as representatives of the three categories based on salt‐gland characteristics, with different concentrations of NaCl under controlled condition. Plant height (Figure 4d), leaf area (Figure 4e), fresh and dry weight (Figure 4f–I) and other biomass of the three types showed a significant decline with increasing NaCl concentration, confirming that salt stress greatly inhibits growth of *Tamarix* (Glenn et al., 1998; Hayes, Walker, & Powell, 2009). However, the response of the three types of *Tamarix* to NaCl stress was different, with type III showing significantly greater inhibition of growth than the other types, including old leaves wilting off. Leaf MDA content also revealed that membrane functions of type III *Tamarix* were harmed by NaCl (Figure 5); however, the MDA contents of type II and type I species showed little change. MDA is a product of membrane lipid peroxidation and can show the degree of peroxidative damage. The lower the amount of MDA produced, the more efficient the plant's antioxidant enzyme system. Different *Tamarix* species have different salt stress tolerances, which leads to great differences in MDA results. According to this parameter, type I *Tamarix* species have evolved the most resistance to salt, while type III species have evolved the least resistance.

*Tamarix* possesses a specific salt‐gland structure, which can secrete excessive ions out of plants. Rising environmental salt content increases the secretion rate of halophyte salt glands (Ma et al., 2011), and secreting excess salt is a mechanism for recretohalophytes to protect themselves from salt stress (Yuan, Xu, Leng, & Wang, 2019). With increasing NaCl concentrations, the mechanism of secreting redundant salt through salt glands is a vital adaptation to saline environments (Helder, 1956; Scholander, 1968; Yuan, Liang, Li, Yin, & Wang, 2019). As illustrated in Figure 6d, the total salt secretion of the three *Tamarix* types was highest under 300 mM NaCl treatment. When NaCl concentration was below 200 mM NaCl, the salt‐gland density of the three *Tamarix* types was greater than that of the control. However, when NaCl concentration reached 300 mM, the density of salt glands decreased, but remained higher than that of the control. At the same time, the secretion rate per salt gland increased steadily with increasing salt concentration, being significantly higher at 300 mM NaCl (Figure 6e). Therefore, we conclude that in the process of *Tamarix* adaptation to salinity, salt glands have evolved in two directions: one to increase the density of salt glands, and the other to increase salt secretion rate per salt gland.

With the increase of salt concentration, the leaf area showed a downward trend, and the density of salt glands showed a trend of first increasing and then decreasing. It is speculated that there are relationships between salt gland density and leaf area. The leaf area decreased at low salt concentration, but the formation of salt glands was not affected, so the density of salt glands increased, and salt‐tolerant varieties still increased at 200 mM salt concentration. High salt concentration also affected leaf area and salt gland formation led to a decrease in leaf area and a decrease in salt gland density.

We observed changes in salt‐gland secretion and structural characteristics in different types of *Tamarix* under salt stress, finding an intrinsic relationship between evolution of salt tolerance and changes in salt glands. We inferred the evolutionary direction of salt glands according to *Tamarix* evolution. A low‐salt environment mainly increases the density of salt glands and thereby the amount of salt excreted to avoid injury. This mechanism is very similar to that of salt tolerance in *Zoysia japonica* Steud (Marcum, Anderson, & Engelke, 1998). When the external salt concentration is very high, increasing the salt secretion rate of a single salt gland is more important for increasing the total salt secretion rate and excreting more salt.

In conclusion, *Tamarix* species can be divided into three types demonstrating salt tolerance in the order type I > type II > type III; this was confirmed by phylogenetic relationships based on ITS sequences. From the comparison of the two clusters in Figure S2, it can be seen that some species are in the same category. The biggest difference is that the third category clustered by some species belongs to the salt‐tolerant species in the ITS evolution tree. But the evolution in the long term also led to some differences.

Salt‐gland evolution has occurred in two directions: one to increase the density of salt glands under low salinity environments, and the other to increase salt secretion rate per salt gland under high salinity environments. However, more research must be conducted using specific molecular markers for salt glands to understand the most reasonable evolution of salt glands.

## CONFLICT OF INTERESTS

None declared.

## AUTHOR CONTRIBUTION

**Xiaocen Wei:** Data curation (lead); Formal analysis (lead); Writing‐original draft (lead); Writing‐review & editing (equal). **Xin Yan:** Data curation (lead); Formal analysis (lead); Investigation (equal); Methodology (equal); Writing‐original draft (lead); Writing‐review & editing (supporting). **Zhen Yang:** Writing‐review & editing (supporting). **Guoliang Han:** Writing‐review & editing (supporting). **Lei Wang:** Data curation (supporting); Writing‐review & editing (supporting). **Fang Yuan:** Writing‐review & editing (lead). **Baoshan Wang:** Methodology (lead); Writing‐review & editing (lead).

## Supporting information

Figure S1

Figure S2

## Data Availability

Evolutionary analysis data are contained in Tables 1 and 2.
