# Supplementary figures and images for: Salt glands of recretohalophyte Tamarix under salinity: Their evolution and adaptation
Source: Ecol Evol. 2020 Aug 11;10(17):9384–95. doi: 10.1002/ece3.6625 (PMC7487237; doi:10.1002/ece3.6625)

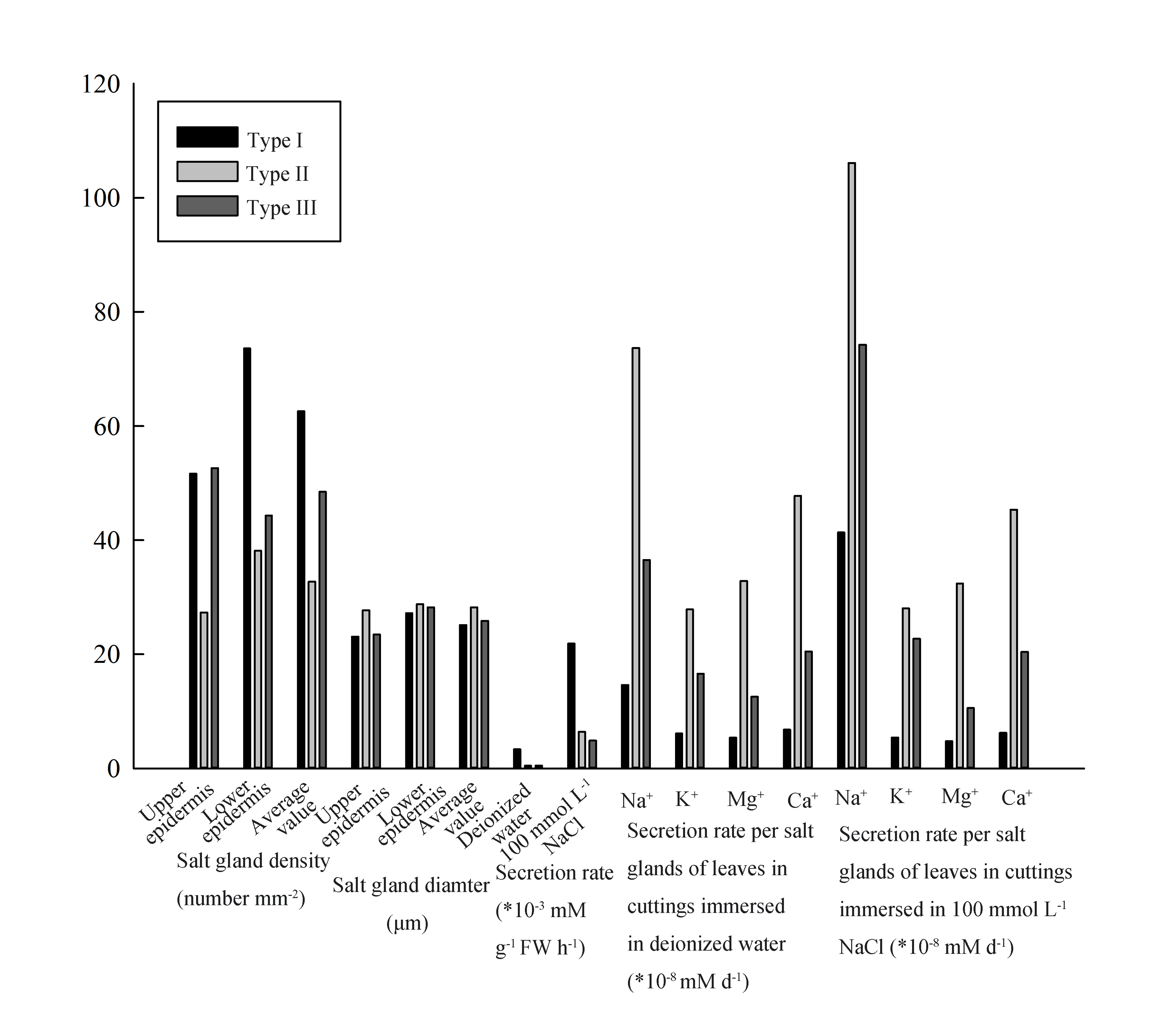

Supplement: Supplementary file 1 — Figure S1 [file ECE3-10-9384-s001.tif]

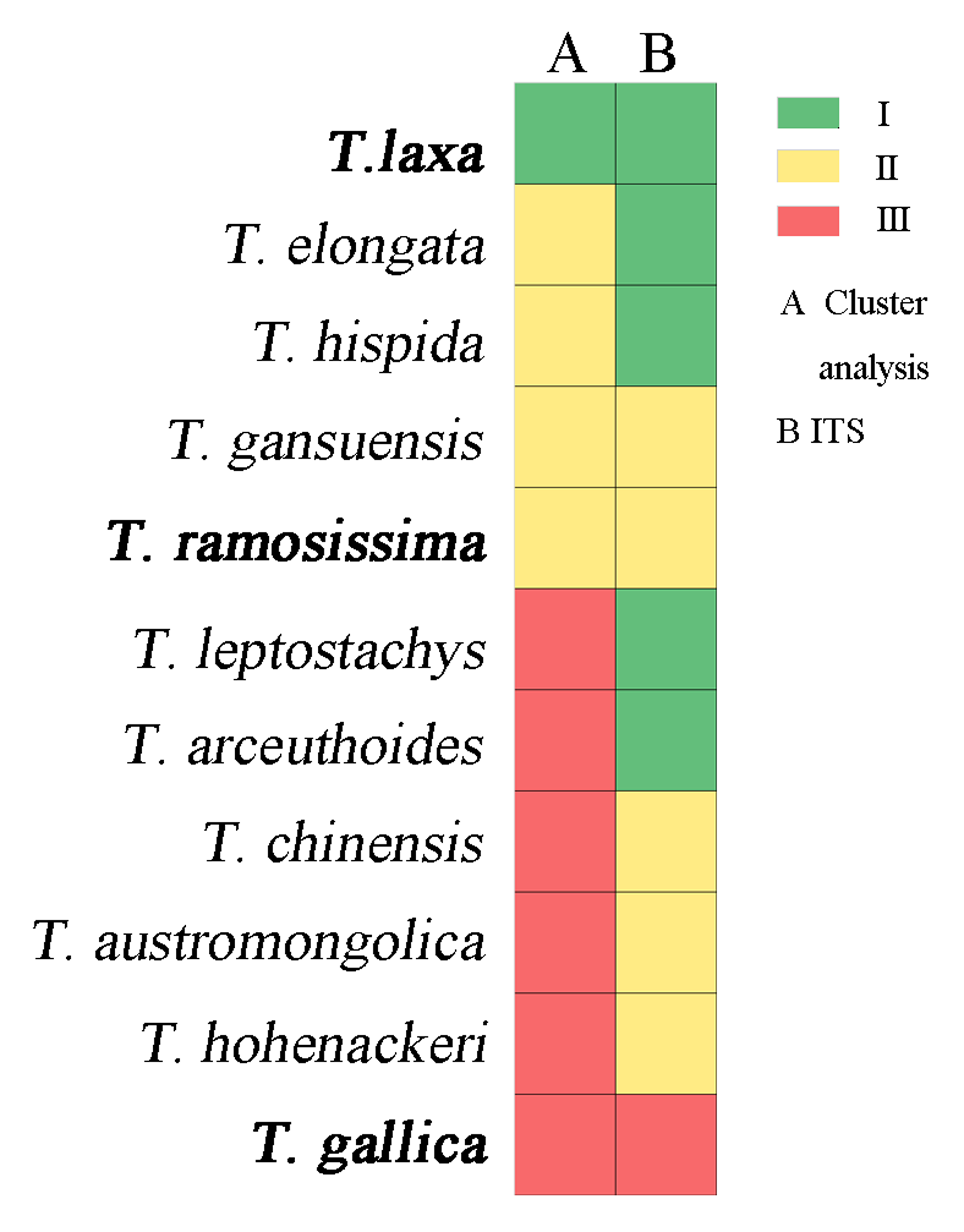

Supplement: Supplementary file 2 — Figure S2 [file ECE3-10-9384-s002.tif]
